# Clinical outcomes of intramedullary nailing for tibial shaft fractures caused by low-energy gunshot versus Gustilo-Anderson type I tibial shaft fractures

**DOI:** 10.1186/s13018-026-06916-y

**Published:** 2026-05-12

**Authors:** Berhan Bayram, Serhat Akcaalan, Tahir Koray Yozgatli, Bulent Tanriverdi, Mustafa Gokhan Bilgili, Baris Kocaoglu

**Affiliations:** 1 Department of Orthopedics and Traumatology, Acıbadem Altunizade Hospital, Istanbul, Türkiye Turkey; 2 Department of Orthopaedics and Traumatology, Ankara City Hospital, Ankara, Türkiye Turkey; 3 Department of Orthopedics and Traumatology, Acibadem MAA University, Istanbul, Türkiye Turkey; 4 Department of Orthopedics and Traumatology, Bakırköy Dr. Sadi Konuk Training and Research Hospital, Istanbul, Türkiye Turkey

**Keywords:** Tibial shaft fracture, Low-energy gunshot injury, Intramedullary nailing, Gustilo-Anderson Type I, Infection

## Abstract

**Background:**

Tibial shaft fractures caused by low-energy gunshot injuries represent a rare but challenging clinical problem. The optimal treatment approach, particularly in comparison with Gustilo-Anderson Type I open fractures, remains controversial.

**Methods:**

This was a retrospective comparative study that included patients who underwent intramedullary nailing for tibial shaft fractures. Group 1 consisted of low-energy gunshot-induced fractures, while Group 2 included Gustilo-Anderson Type I open fractures. Demographic data, union time, Radiographic Union Score for Tibial Fractures (RUST), Johner-Wruhs functional classification, need for additional surgical intervention, and infection rates were compared.

**Results:**

A total of 128 patients were included (68 in Group 1 and 60 in Group 2). No significant differences were observed between the groups regarding demographic characteristics, follow-up duration, union time, RUST scores, or Johner–Wruhs functional outcomes (*p* > 0.05). However, the infection rate was significantly higher in the low-energy gunshot group compared with the Gustilo–Anderson type I group (20.6% vs. 5.0%, *p* = 0.020).Nonunion and reoperation rates did not differ significantly between the groups.

**Conclusion:**

Intramedullary nailing for tibial shaft fractures caused by low-energy gunshot injuries provides clinical and radiological outcomes comparable to those observed in Gustilo–Anderson type I open fractures. Nevertheless, these injuries appear to be associated with a higher risk of infection, warranting careful perioperative management and closer postoperative surveillance.

**Supplementary Information:**

The online version contains supplementary material available at 10.1186/s13018-026-06916-y.

## Introduction

Tibial shaft fractures are common long bone fractures encountered in daily practice, and open tibial shaft fractures constitute an important subgroup of these fractures [1]. Tibial shaft fractures account for 36.7% of long bone fractures and more than 2% of all fractures [1, 2]. Since the tibia is covered with only a thin layer of soft tissue, more than 15% of tibial fractures are open fractures [2]. The Gustilo-Anderson classification is one of the most commonly used systems for determining the severity and treatment of open fractures. This classification groups open fractures into Types I, II and III (subtypes A, B, C) based on wound size, soft tissue injury, degree of contamination and ruptures such as vascular injuries. This approach guides both prognosis and surgical treatment decisions [3].

The incidence of firearm injuries is increasing significantly due to the increasing trend of violence in societies worldwide [4]. The treatment of gunshot wounds can be challenging for orthopedists because these injuries can involve various structures such as soft tissues, bone, muscle-tendon, vascular structures, and nerves. The majority of gunshot wounds in long bones are associated with comminuted open fractures. Different treatment options such as splinting, external fixation, internal fixation, and intramedullary nailing may be considered. Low-energy gunshot wounds are usually caused by low-velocity weapons, such as handguns, and result in limited fractures with more localized tissue damage. On the other hand, high-energy injuries are caused by high velocity weapons such as rifles and are characterized by bone fragmentation and extensive soft tissue damage with a higher risk of infection and complications [5].

Intramedullary nailing is the preferred treatment method for tibial shaft fractures because it provides rapid bone healing and early weight-bearing [6, 7]. Early clinical studies have also demonstrated favorable outcomes of interlocking intramedullary nailing for long bone fractures of the femur and tibia [8]. However, the treatment approaches in open tibial shaft fractures and gunshot wounds are unclear and controversial [9].

The aim of the current study was to compare the clinical and radiologic outcomes of Gustilo Anderson Type 1 tibia shaft open fractures and tibia shaft fractures due to low-energy gunshots treated with intramedullary nailing. Although low-energy gunshot injuries are often considered equivalent to Gustilo–Anderson type I open fractures, differences in injury mechanism and soft tissue contamination may influence clinical outcomes. Therefore, we aimed to compare these two groups separately to better understand whether low-energy gunshot fractures truly behave similarly to Gustilo–Anderson type I injuries in terms of clinical and radiological outcomes. The hypothesis was that the clinical and radiologic outcomes of tibia shaft fractures due to low-energy gunshot injury and Gustilo Anderson Type 1 tibia shaft open fractures would be similar.

## Materials and methods

This was a retrospective comparative study. Data collection for the study was started after the ethics committee approval was obtained (IRB Approval No: 2025-01-09; Protocol No: 2025/334; Approval Date: 24 October 2025). The tibial shaft fractures were analyzed in two groups. Isolated tibial shaft fractures caused by low-energy gunshot wounds (Group 1) and Gustilo Anderson type 1 open fractures (Group 2) were included in the study. Low-energy gunshot injuries were defined as civilian gunshot wounds caused by low-velocity firearms without extensive soft-tissue destruction. Because detailed ballistic parameters such as projectile velocity or firearm type were not available in the medical records, the classification was based on clinical injury characteristics consistent with low-velocity civilian gunshot trauma. Inclusion criteria for the low-energy gunshot group included limited soft-tissue damage, absence of segmental bone loss, and wound characteristics consistent with low-velocity projectile injury. Fractures with extensive soft-tissue damage, segmental bone loss, or features suggesting high-energy ballistic trauma were excluded from the study. Patients with Gustilo Anderson type 2–3 fractures, tibial shaft fractures caused by high-energy gunshot wounds, patients with additional system injuries, patients with a history of previous trauma or surgery on the same extremity, patients with multiple fractures, patients with neurovascular injury in the involved extremity, patients with a history of neurovascular pathology in the involved extremity, and patients without regular clinical and radiologic follow-up were excluded.

At initial diagnosis of an open tibial shaft fracture, all patients underwent a standardized intervention in the emergency department, which consisted of irrigation and debridement of the wound followed by long leg splinting of the injured extremity. Intravenous third-generation cephalosporin was administered in the emergency department as part of the institutional trauma protocol and continued for at least 24 h during the hospital stay, followed by transition to first-generation cephalosporin in the postoperative period. Tetanus prophylaxis was administered to eligible patients by reviewing their medical history. All patients underwent surgery within 6 h of the first intervention. All surgeries were performed in a single center and by a single senior surgeon. All patients underwent spinal anesthesia, and surgeries were performed in the supine position without tourniquet application. After appropriate preparation and sterile draping, a wound debridement was performed again. All open fractures, including both low-energy gunshot injuries and Gustilo–Anderson type I fractures, were managed using the same standardized debridement protocol. Initial irrigation and debridement were performed in the emergency department, followed by a second formal debridement in the operating room prior to definitive fixation. In both groups, wounds were extended when necessary to allow adequate visualization, and devitalized soft tissue and contaminated bone fragments were meticulously debrided. Particular attention was paid to removing all non-viable tissue and minimizing bacterial contamination.There was no difference in the debridement principles applied between the two groups. All procedures were performed by the same senior surgeon using a consistent open fracture management protocol. Following debridement a standardized intramedullary nailing surgery was performed. All nails were inserted after appropriate intramedullary reaming. The medullary canal was reamed to the appropriate diameter, and nails of suitable size were implanted. Nail locking was performed both proximally and distally in accordance with the fracture type.

In the postoperative period, all patients were followed with intravenous antibiotics for the duration of hospital stay and were encouraged to initiate weight-bearing as tolerated, based on pain and compliance. All patients received postoperative prophylaxis with a first-generation cephalosporin (e.g., cefazolin) for a total duration of 72 h. This protocol was based on evidence demonstrating that short-course first-generation cephalosporin prophylaxis (24–72 h) is sufficient for Gustilo–Anderson type I open fractures, with no additional benefit of prolonged antibiotic administration in reducing infection rates [3, 10, 11]. Furthermore, civilian low-energy (low-velocity) gunshot–related tibial shaft fractures with minimal soft-tissue injury have been shown to behave similarly to Gustilo–Anderson type I open fractures, and can therefore be managed using comparable antibiotic and surgical principles [9, 12]. Accordingly, patients in both groups were followed using the same postoperative antibiotic regimen in our institution. No differences in antibiotic selection, duration of therapy, or wound management strategy were applied between the two study groups.Patients were discharged from the hospital with appropriate pain medication and followed up regularly in the 2nd week, 1st month, 2nd month, 3rd month, 6th month, 1st year. Then annual follow-ups were started. Dynamization was applied if there was not enough improvement in the 3rd month post-operatively.

Demographics such as age and gender of all patients in the study were recorded. Follow-up durations, union time, additional surgical interventions, and presence of infection were recorded separately for all patients. Infection was classified as superficial or deep infection based on clinical findings and treatment requirements. Superficial infection was defined as localized erythema, drainage, or wound inflammation requiring oral antibiotic therapy without surgical intervention. Deep infection was defined as infection requiring surgical debridement, intravenous antibiotic therapy, or implant-related management. The diagnosis of infection was based on clinical evaluation, laboratory findings, and intraoperative assessment when applicable. Infections were recorded during the postoperative follow-up period and were considered present if diagnosed at any time until fracture union or the final follow-up visit.Union was evaluated by two different orthopedic surgeons with more than ten years in trauma surgery. Fracture union was primarily assessed using the Radiographic Union Score for Tibial Fractures (RUST). In this scoring system, the four cortices of the tibia were scored separately (1 = no callus, 2 = callus present but fracture line visible, 3 = complete union), and the total score ranged from 4 to 12, with higher scores indicating a more advanced degree of healing. RUST scoring was performed using serial radiographs obtained during follow-up [RUST ref].Union time was defined as the time from surgery to the first radiographic evidence of union based on RUST assessment. Clinical evaluation, including the ability to bear weight and walk without pain, was used as a supportive parameter but was not considered the primary outcome measure [9, 13].

Functional outcomes were evaluated using the Johner-Wruhs classification. This classification includes parameters such as pain, walking capacity, knee and ankle range of motion, angular deformity, shortness, and complications and the results were evaluated as excellent, good, fair, and poor and recorded separately for each patient [14]. Johner–Wruhs functional evaluation was performed at the final follow-up visit.

**Fig. 1:**
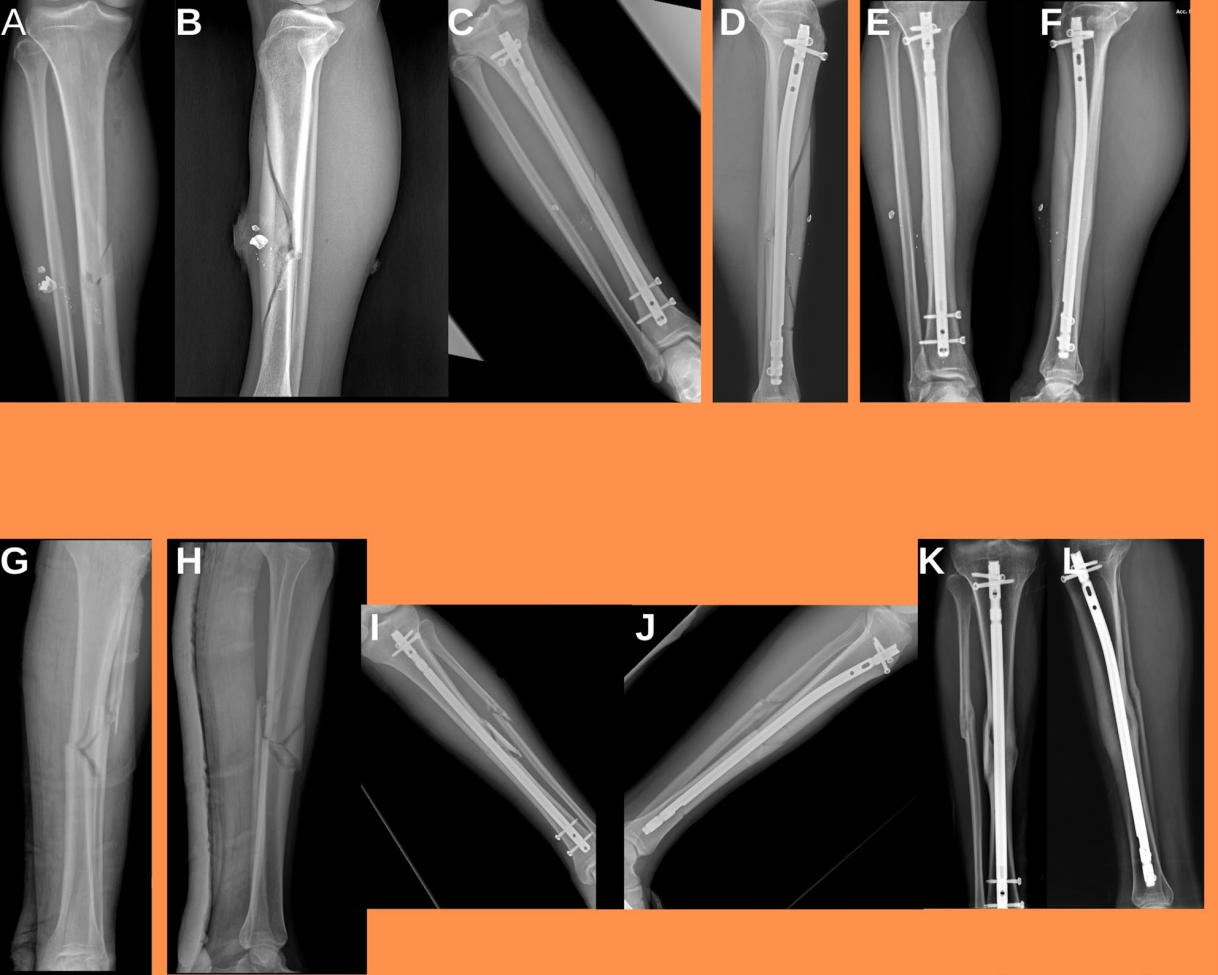
Representative radiographic images of tibial shaft fractures treated with intramedullary nailing in both study groups. **A**–**F** A representative case of a tibial shaft fracture caused by a low-energy gunshot injury: preoperative anteroposterior and lateral radiographs demonstrating fracture pattern and retained bullet fragments, followed by immediate postoperative and follow-up radiographs showing intramedullary nail fixation and fracture healing. **G**–**K** A representative case of a Gustilo–Anderson type I open tibial shaft fracture: preoperative anteroposterior and lateral radiographs, postoperative images after intramedullary nailing, and follow-up radiographs demonstrating fracture union. These images illustrate the comparable radiological outcomes achieved with intramedullary nailing in both low-energy gunshot–related and Gustilo–Anderson type I tibial shaft fractures

Representative preoperative, postoperative, and follow-up radiographs illustrating intramedullary nailing in both low-energy gunshot–related and Gustilo–Anderson type I tibial shaft fractures are presented in Fig. 1.

### Statistical analysis 

Frequency and percentage (n(%)) statistics were given for categorical (qualitative) variables, and mean, standard deviation (mean ± ss), minimum, maximum, and median (M) statistics were given for numerical (quantitative) variables. The conformity of continuous variables to the normal distribution was assessed with the calculation of skewness and kurtosis. The kurtosis and skewness values obtained from the measurements between + 3 and − 3 were considered sufficient for normal distribution [15]. Both parametric and nonparametric methods were used in the analyses depending on the distribution. Independent groups t/Mann Whitney test was used in the comparison of the numerical variables determined in the study according to the groups and Chi-square test was used in the relationships between grouped variables. Data analysis was performed using SPSS 27.0. In addition to p-values, effect size estimates were calculated as risk ratios (RR) with corresponding 95% confidence intervals. In addition to p-values, effect size estimates were calculated as risk ratios (RR) with corresponding 95% confidence intervals for categorical outcomes.

## Results 

There were 314 tibial shaft fractures fixed with intramedullary nails between 2012 and 2018. Of the initial 314 patients, 186 were excluded based on the predefined criteria. A total of 71 patients were excluded due to Gustilo–Anderson type II–III fractures, 19 due to high-energy gunshot injuries, 22 due to additional system injuries, 20 due to a history of previous trauma or surgery in the same extremity, 12 due to multiple fractures, 11 due to neurovascular injury, and 31 due to incomplete clinical or radiological follow-up. The remaining 128 patients met the inclusion criteria and were included in the final analysis.After exclusion criteria, a total of 128 patients (68 in Group 1 and 60 in Group 2) were included in the study. Baseline demographic and clinical characteristics of the patients are summarized in Table 1. The gender distributions were similar between groups (Group 1: 47 males, 69.1% vs. Group 2: 43 males, 71.7%, *p* = 0.904). The ages were similar between the groups (Group 1: 33.57 ± 8.30 years vs. Group 2: 35.70 ± 8.26 years, *p* = 0.150). Follow up periods were similar between the groups (Group 1: 31.40 ± 14.17 months vs. Group 2: 32.03 ± 14.01 months, *p* = 0.901). The overall mean follow-up duration for the entire cohort was 31.70 ± 14.10 months. Table 2 shows the union times, RUST score and Johner-Wruhs classification of the patients in the study. According to the Johner–Wruhs classification, the majority of patients in both groups achieved excellent or good outcomes. In Group 1, 44 patients (64.7%) had excellent results and 23 patients (33.8%) had good results, while in Group 2, 43 patients (71.7%) had excellent results and 13 patients (21.7%) had good results. Fair outcomes were observed in 1 patient (1.5%) in Group 1 and 4 patients (6.7%) in Group 2. There was no statistically significant difference between the groups (*p* = 0.149).

**Table 1:** Baseline demographic characteristics of the study groups

Variable	Group 1 (*n* = 68)	Group 2 (*n* = 60)	*p *value
Sex (Male/Female)	47 / 21	43 / 17	0.904
Age (years), mean ± SD	33.57 ± 8.30	35.70 ± 8.26	0.150
Follow-up duration (months), mean ± SD	31.40 ± 14.17	32.03 ± 14.01	0.901

**Table 2:** Union time, RUST score, and Johner–Wruhs functional outcomes in Group 1 and Group 2

Parameters		Group 1	Group 2	*P* values
Union time (weeks)		22.72 ± 5.21	22.52 ± 4.80	0.819
RUST score		10.24 ± 1.44	10.58 ± 1.17	0.138
Johner–Wruhs classification	Fair	1 (%1.5)	4 (%6.7)	0.149
	Good	23 (%33.8)	13 (%21.7)	
	Excellent	44 (%64.7)	43 (%71.7)	

Postoperative complication outcomes are summarized in Table 3. Infection rates were significantly higher in Group 1 compared with Group 2 (14/68, 20.6% vs. 3/60, 5.0%; *p* = 0.020). The calculated risk ratio was 4.12 (95% CI 1.22–13.90).Nonunion rates were similar between groups (14/68, 20.6% vs. 10/60, 16.7%; *p* = 0.734). Secondary operations were required in 9 patients (13.2%) in Group 1 and 12 patients (20.0%) in Group 2 (*p* = 0.428).In Group 1, 6 patients underwent dynamization and no patients underwent nail exchange. In Group 2, 7 patients underwent dynamization and one patient underwent nail exchange.

**Table 3:** Postoperative complications

Outcome	Group 1 (*n* = 68)	Group 2 (*n* = 60)	*p *value
Infection	14 (20.6%)	3 (5.0%)	0.020
Nonunion	14 (20.6%)	10 (16.7%)	0.734
Reoperation	9 (13.2%)	12 (20.0%)	0.428
Dynamization	6	7	–
Nail exchange	0	1	–

## Discussion 

The most important finding of this study was that intramedullary nailing of tibial shaft fractures caused by low-energy gunshot wounds showed similar clinical and radiologic results to Gustilo Anderson Type 1 open fractures, but had a higher infection rate.

Previously, Lee et al. investigated the rate of complications in low-energy gunshot-induced tibia fractures [16]. They reported that the overall complication rate was as high as 49%, of which 14% was infection, 27% was wound complications and 20% was nonunion. The fact that such high complication rates are seen in the literature reveals how important the choice of treatment is in such cases. Similarly, Yeganeh et al. evaluated the results of 50 patients admitted to their clinic with open gunshot fractures of the tibia, humerus and femur and emphasized that problems such as infection and nonunion can be seen at a high rate, so the process should be managed more carefully [17]. In the current study, the overall infection rate was 13.3% (17/128). Infection occurred in 14 patients (20.6%) in the low-energy gunshot group and in 3 patients (5.0%) in the Gustilo–Anderson type I fracture group. The calculated risk ratio was 4.12 (95% CI 1.22–13.90), indicating a higher infection risk in the low-energy gunshot group. Despite this difference in infection rates, union time, RUST scores, and functional outcomes were comparable between the two groups. The discrepancy between higher infection rates and similar reoperation rates between groups may be explained by the nature of infections observed in this study. Not all infections required surgical intervention, as a proportion of cases were classified as superficial infections and were successfully managed with oral antibiotic therapy alone. Therefore, the higher infection rate in the low-energy gunshot group did not necessarily translate into a higher reoperation rate. In addition, none of the patients in this cohort required extensive reconstructive procedures such as segmental bone resection, defect reconstruction, or soft tissue flap coverage due to infection. When surgical intervention was necessary, it was limited to relatively minor procedures such as debridement or dynamization. These findings support that the majority of infections observed in this study were of limited severity.The relatively high infection rates observed in both groups in the present study may be explained by several factors. First, the infection endpoint in this study included both superficial and deep infections based on clinical findings and treatment requirements, which may lead to higher reported rates compared with studies reporting only deep infections or fracture-related infections. Second, host-related factors such as smoking status, comorbidities, and patient-specific characteristics may also influence infection risk, although these variables could not be consistently evaluated due to the retrospective design of the study. Third, even low-energy gunshot injuries may introduce bacterial contamination into deep tissues despite limited soft-tissue damage. Finally, variations in institutional treatment protocols, wound management strategies, and postoperative surveillance practices may also contribute to differences in reported infection rates across studies.

Pinto et al. evaluated the effectiveness of gentamicin-coated intramedullary nails and regular nails in Gustilo Type 1 and 2 fractures and showed that gentamicin-coated intramedullary nails were more effective in decreasing infection rates [11]. Donnally et al. evaluated the results of the patient group in which they applied intramedullary nails in low-velocity gunshot tibia fractures and stated that formal debridement followed by intramedullary nailing in low-velocity gunshot tibia fractures may increase the risk of superficial infection but not the risk of deep infection [12]. In the current study, all patients in Group 1 and Group 2 underwent formal debridement, and overall infection rates were similar to the literature. Su et al. also shared the results of patients treated for tibia fractures caused by low-engery gunshot. Su et al. stated that the infection rate of these injuries was not very high and they did not encounter non-union [18].They showed that the infection and non-union rates were similar to those of tibia shaft fractures caused by blunt trauma [18]. In contrast, in the current study, the gunshot-wound group had a higher infection rate. The higher infection rate in Group 1 may be due to the gunshot wound, which causes bacterial contamination of deep tissues.

Metcalf et al. compared tibia shaft fractures caused by blunt trauma with tibia shaft fractures caused by gunshot wounds and emphasized that the infection rates were similar, but the results may be more related to fragmentation in the bone [19]. Ateş et al. shared the data of 124 patients treated for gunshot wounds and emphasized that the severity of the injury was related to the complication rate regardless of the anatomical localization and treatment method [20]. Thus, there are contradicting results in the literature regarding the treatment outcomes of patients with long bone fractures due to gunshot wounds. In recent years, increasing attention has been directed toward fracture-related infection (FRI) prevention and management in open tibial fractures. Contemporary orthopaedic trauma research has emphasized standardized definitions of FRI, improved infection risk stratification, and the development of modern infection prevention strategies. In particular, antibacterial-coated intramedullary nails and optimized perioperative protocols have been investigated as potential strategies to reduce infection risk in open fractures [21, 22]. These developments highlight the importance of careful infection surveillance and individualized treatment strategies in patients with open tibial shaft fractures. In the current study, the radiologic and clinical outcomes of low-energy gunshot tibial shaft fractures and Gustilo Anderson Type 1 open fractures treated with intramedullary nailing were compared. The results showed that tibial shaft fractures caused by low-energy gunshot wounds had a higher risk of infection only, and there was no difference in terms of other radiologic and clinical variables. Despite similar outcomes in terms of union and function, surgeons should be aware of the increased infection rate that may be associated with tibial shaft fractures caused by gunshot wounds and treated with intramedullary nailing.

Bauwens et al. identified risk factors for complications in their study of 184 patients with tibial shaft fractures treated with intramedullary nailing. According to the data in this study; active smoking, a residual interfragmentary gap > 5 mm and open fracture were determined as risk factors [23]. Thus it should be kept in mind that these subgroup of patients may be more likely to develop infection.

This study is not without limitations. First, its retrospective design inherently limits the ability to establish causal relationships. Second, the sample size, although adequate for statistical analysis, may not fully represent the heterogeneity of tibial gunshot injuries. Third, the study was conducted in a single center by a single surgeon, which may limit the external validity and generalizability of the findings. In addition, although the study covered a relatively long retrospective period (2012–2018), the institutional treatment protocol for open tibial shaft fractures, including antibiotic prophylaxis and surgical technique, remained unchanged throughout the study period. Fourth, due to the retrospective design of the study, some potentially relevant confounding variables such as smoking status, diabetes or other comorbidities, body mass index, fracture location characteristics, and time to antibiotic administration were not consistently recorded in the medical records and therefore could not be included in the analysis. Finally, the absence of long-term functional outcome measures beyond the Johner-Wruhs classification represents another limitation. Future prospective multicenter studies with larger cohorts are needed to validate these results.

In conclusion, debridement and primary intramedullary nailing of tibial shaft fractures due to low-energy gunshot wounds provide similar clinical and radiologic results to Gustilo-Anderson Type I fractures. However, this approach poses a greater risk of infection. Especially for patients defined in the literature as high-risk for complication development, more careful, close follow-up may be required after surgical treatment. These findings suggest that although intramedullary nailing remains a viable treatment option for low-energy gunshot–related tibial shaft fractures, surgeons should remain vigilant regarding infection risk and consider appropriate perioperative infection prevention strategies.

## Supplementary Information

Below is the link to the electronic supplementary material.

Supplementary Material 1

## Data Availability

The datasets used and/or analyzed during the current study are available from the corresponding author on reasonable request.
